# Incomplete penetrance of a novel SDHD variation causing familial head and neck paraganglioma

**DOI:** 10.1111/coa.13782

**Published:** 2021-05-05

**Authors:** Martin Koenighofer, Thomas Parzefall, Alexandra Frohne, Elisabeth Frei, Christian Schoefer, Franco Laccone, Patricia Feil, Klemens Frei, Trevor Lucas

**Affiliations:** ^1^ Department of Otorhinolaryngology, Head and Neck Surgery Medical University of Vienna Vienna Austria; ^2^ Center of Anatomy and Cell biology Medical University of Vienna Vienna Austria; ^3^ Department of Medical Genetics Medical University of Vienna Vienna Austria; ^4^ Department of Pediatric Surgery Medical University of Vienna Vienna Austria

**Keywords:** familial 1, frameshift variation, head and neck neoplasms, orphan diseases, paragangliomas, paternal inheritance, succinate dehydrogenase

## Abstract

**Objective:**

Identification of variations in tumour suppressor genes encoding the tetrameric succinate dehydrogenase (SDHx) mitochondrial enzyme complex may lead to personalised therapeutic concepts for the orphan disease, familial paraganglioma (PGL) type 1‐5. We undertook to determine the causative variation in a family suffering from idiopathic early‐onset (22 ± 2 years) head and neck PGL by PCR and Sanger sequencing.

**Design:**

Prospective genetic study.

**Setting:**

Tertiary Referral Otolaryngology Centre.

**Participants:**

Twelve family members.

**Main outcome measures:**

Main outcomes were clinical analysis and SDH genotyping

**Results and Conclusions:**

A novel heterozygous c.298delA frameshift variation in exon 3 of SDH subunit D (SDHD) was associated with a paternal transmission pattern of PGL in affected family members available to the study. Family history over five generations in adulthood indicated a variable penetrance for PGL inheritance in older generations. The c.298delA variant would cause translation of a 34‐residue C‐terminus distal to lysine residue 99 in the predicted transmembrane domain II of the full‐length sequence p.(Thr100LeufsTer35) and would affect the translation products of all protein‐coding SDHD isoforms containing transmembrane topologies required for positional integration in the inner mitochondrial membrane and complex formation. These results underly the importance of genetic screening for PGL also in cases of unclear inheritance, and variation carriers should benefit from screening and lifelong follow‐up.

**Keypoints**

- An Austrian Caucasian family was identified segregating late‐onset head and neck paraganglioma (hnPGL) over four generations.
- Variations in genes, encoding the tetrameric succinate dehydrogenase (*SDH*) II mitochondrial enzyme complex, were examined by PCR and Sanger sequencing.
- A novel *SDHD* frameshift variation in exon 3 was isolated showing paternal transmission.
- The c.298delA variation (p.Thr100LeufsTer35) would affect the translation products of all protein‐coding SDHD isoforms containing transmembrane topologies.
- Genetic testing is highly recommended in cases of hnPGL. Patients with a SDHD variation should benefit from complete clinical screening and lifelong follow‐up.

## 1 INTRODUCTION

Paragangliomas (PGL) are neuroendocrine tumours of the peripheral nervous system arising in cells derived from the neural crest. PGL are rare and occur with an estimated incidence of 1‐9 cases per million persons per year and are registered as an orphan disease by the U.S. Office of Rare Diseases (GARD) and European Orphanet (classification number ORPHA29072).

PGL are classified as adrenal PGL originating in the adrenal medulla as pheochromocytoma (PCC) or extra‐adrenal PGL that may occur in the abdomen, thorax and head and neck regions.^1^ The most common locations are carotid, jugulotympanic and vagal head and neck (hn)PGL. Very rarely laryngeal PGL can occur.^2^ Although PGL at any site can release catecholamines, this is more common for adrenal tumours and approximately 95% of hnPGL are nonsecretory.^3^

A genetic cause can be found in approximately 40% of hnPGL patients, carrying a variation in one of more than 20 genes.^4^ Around half of PGL and PCC cases show germline variations in the succinate dehydrogenase complex genes (*SDHx*)^4^, ^5^, ^6^ and are classified as PGL syndromes PGL1‐5 (table 1). PGL1 (OMIM #168000) is caused by variations in the succinate dehydrogenase complex subunit D gene (*SDHD*, OMIM *602690*)*.^7^ SDHD variants cause over 97% of cases of hnPGL^4^ summarised in table S1.

**TABLE 1:** Reported SDHD mutations and total numbers of cases published

Familial paraganglioma	OMIM#	Gene	OMIM*	Genomic locus	Percentage of hnPGL	Transmission
PGL1	168000	*SDHD*	602690	11q23.1	59% (1)	Paternal (AD)
PGL2	601650	*SDHAF2*	613019	11q12.2	Unknown	Paternal (AD)
PGL3	605373	*SDHC*	602413	1q23.3	5% (2)	AD
PGL4	115310	*SDHB*	185470	1p36.13	12% (3)	AD
PGL5	614165	*SDHA*	600857	5p15.33	1.8% (4)	AD

Note

Inheritance of familial paraganglioma (PGL). PGL is caused by variations in mitochondrial complex II subunits *SDHD*, *SDHC*, *SDHB* and *SDHA* and the regulatory subunit succinate dehydrogenase 5 (*SDHAF2*). Inheritance is autosomal dominant (AD) and predominantly paternal in PGL1‐2 (^*^phenotype, ^#^gene/locus).

Furthermore, PGL1 patients show a high occurrence of multifocal and reoccurring tumours in contrast to patients with causative variations in other SDHx genes.^8^, ^9^, ^10^, ^11^ The mean onset of disease in familial hnPGL is below 45 years of age.^8^, ^9^, ^10^, ^12^ The transmission in PGL1 patients demonstrates a parent‐of‐origin effect and is mostly transmitted paternally^5^, ^10^, ^13^, ^14^, ^15^ but also maternally.^16^, ^17^, ^18^, ^19^ Both loss of heterozygosity at 11q22‐23^20^ and somatic loss of the entire maternal chromosome 11 have been observed in 85% of SDHD caused PGL.^21^

SDHD is the smallest unit of the succinate dehydrogenase (SDH) complex located in the mitochondrial membrane and involved in both the Krebs cycle and the respiratory chain catalysing the oxidation of succinate to fumarate coupled with the reduction of ubiquinone to ubiquinol. PGL tumourigenesis is believed to be initiated through the accumulation of succinate and reactive oxygen species (ROS) due to reduced SDH complex efficiency, increased reliance on glycolysis, protection against apoptosis and triggering a chronic pseudo‐hypoxic response including increased angiogenesis. ^7^, ^22^

In SDHD variation carriers, this abnormal vascularisation may increase the risk of ischaemic stroke following preoperative embolisation.^23^ Succinate accumulation can also inhibit α‐ketoglutarate‐dependent dioxygenases leading to large‐scale hypermethylation and epigenetic reprogramming induced by inhibition of DNA and histone demethylases.^24^

Here we identify a novel pathogenic, paternally inherited *SDHD* variant with incomplete penetrance in a non‐consanguineous hnPGL family of Austrian descent. Knowledge of the cause of disease allows early intervention to monitor any development of PGL in a personalised medicine approach.^25^, ^26^

## 2 MATERIALS AND METHODS

### 2.1 Subjects

Patients were recruited from the Department of Otorhinolaryngology, Head and Neck Surgery at the Medical University of Vienna hospital. Detailed medical and family histories were taken, and complete otorhinolaryngological examination was performed where possible. Diagnosis of hnPGL was made from medical records of a generation II patient and a generation III sufferer not available to the study. Magnetic resonance imaging (MRI) was performed with a 3 Tesla TRIO 32 canal machine with multicore capability (Siemens) with macro‐ and micro‐catheters and selective embolisation of tumour feeders with polyvinyl ethanol particles for angiography (150 μm). In addition to standard analysis, in patients (III.2, IV.1 and V.4) and unaffected variation carriers (IV.3, IV.4, V.5 and V.6), 6‐Fluoro‐(18F)‐l‐3,4‐dihydroxyphenylalanine (18F‐DOPA) positron emission tomography (PET) scans or thoracic/abdominal computerized tomography (CT) scans were performed. If blood pressure levels were elevated, metanephrine values were assessed.

### 2.2 Ethical considerations

Informed consent was obtained prior to study inclusion. The trial was approved by Medical University of Vienna ethics committee (1749/2014) and was performed in accordance with ethical standards laid down in the 1964 Declaration of Helsinki.

### 2.3 DNA isolation, PCR and bioinformatics

Molecular testing was performed retrospectively after surgical treatment. DNA was extracted from peripheral blood with the Invisorb blood universal kit 1000 (Stratec Molecular). All *SDHB* (NM_003000.2/ENST00000375499), *SDHC* (NM_003001.5/ENST00000367975) and *SDHD* (NM_003002.4/ENST00000375549) reference sequence coding exons including flanking intronic regions were amplified and sequenced in the index patient. The c.298delA *SDHD* variant was screened in all available family members by sequencing a 348bp amplification product generated with exon 3 specific forward 5′TGCCTGTCAGTTTGGGTTACTGTG‐3′ and reverse 5′‐TCAACAAATTTAGGGCATTTCAATC‐3′ primers. The c.298delA variant was registered at ClinVar (ncbi.nlm.nih.gov/clinvar) as SCV000575875. Splice variant coding sequences were the 120 residue *SDHD‐202* (NM_001276504.2/ENST00000525291.5) and 143 residue *SDHD‐003* (ENST00000528048/NM_001276503).

Genes were analysed at the Ensembl database (ensembl.org/index.htmlHuman), the Human Gene Mutation database (hgmd.cf.ac.uk/ac/index.php), dbSNP (ncbi.nlm.nih.gov/SNP), the gnomAD database from the Exome Aggregation Consortium (gnomad.broadinstitute.org, version 2.1.1)^27^, ClinVar (ncbi.nlm.nih.gov/clinvar), the Leiden Open Variation Database v.3.0 (lovd.nl) and the UniProt Knowledgebase (UniProtKB; uniprot.org). The identified variant was classified with Mutalyzer (https://mutalyzer.nl) and analysed with MutationTaster (mutationtaster.org).

## 3 RESULTS

**FIGURE 1:**
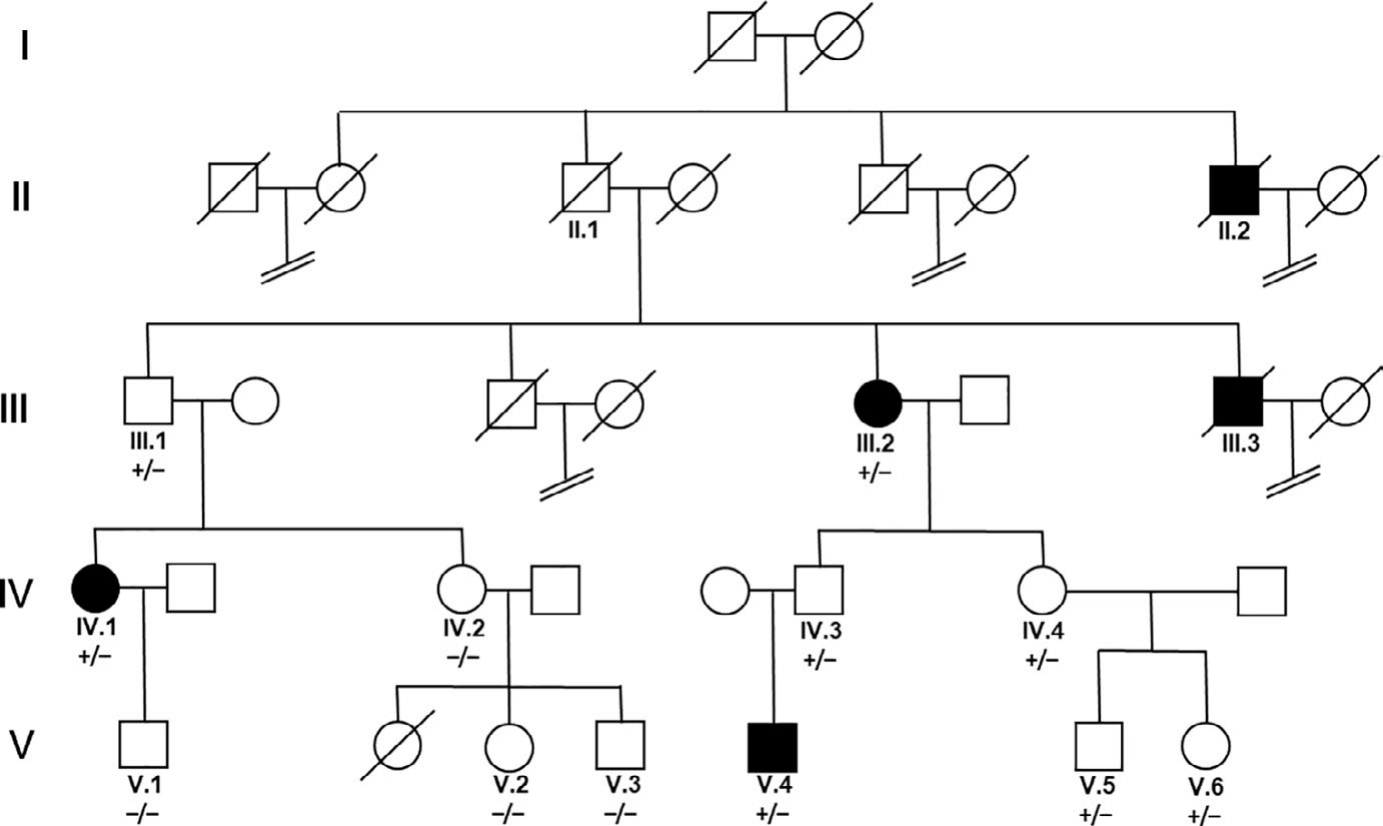
Inheritance of head and neck paraganglioma (shaded patients) in the family under study. The index patient was V.4. The heterozygous c.298delA *SDHD* variation is carried (+/‐) and transmitted by females but only manifests as disease when paternally inherited in IV.1 and V.4. In generation II and family member III.1 (91 y of age), low penetrance is observed

**FIGURE 2:**
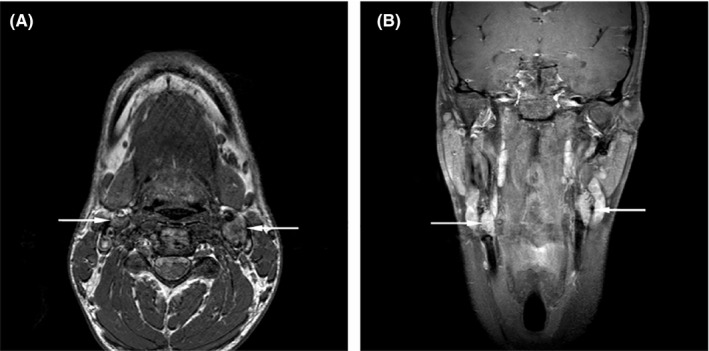
Magnetic resonance imaging (T1 weighting) of a 31‐year‐old male (family member V.4) diagnosed with bilateral, carotid hnPGL shown in axial A, and coronal B, views (arrowed)

In a non‐consanguineous Caucasian family from Austria (population 8.5 million), patients (n = 3/12) were diagnosed with hnPGL with a median age of onset of 22 ± 2 years (Figure 1). HnPGL patients included in the study were tumour‐resected. Proband II.1 died at the age of 82 with no signs of hnPGL detectable by neck ultrasound imaging. Patient II.2 was diagnosed with a neck lump suggestive of hnPGL but died in 1903 at age 26 of other causes. The 88‐year‐old affected patient (III.2) with an asymptomatic brother (III.1) was diagnosed at the age of 20 with a unilateral, right‐sided carotid hnPGL, which was resected 2 years after diagnosis. Patient III.2 died of other causes than hnPGL at the age of 90. At the age of 62, patient III.3 underwent removal of an intracranial PGL followed by radiation therapy following prior surgical excision of a carotid hnPGL. Patient III.3 died at the age of 80 due to cachexia caused by malignant melanoma. The affected 56‐year‐old family member (IV.1) was diagnosed with right‐sided jugulotympanic hnPGL and left‐sided carotid and vagal hnPGL at 22 years of age. Resection of the jugulotympanic hnPGL was performed at 34 years of age, and resection of the left‐sided tumours was performed a year later. The 31‐year‐old male index patient (V.4) was diagnosed at the age of 24 with bilateral carotid hnPGL confirmed by MRI (figure 2) with a follow‐up of approximately 11 years. Angiography showed vascularisation from the ascending pharyngeal artery and the external carotid artery. Tumour resection was performed in the same year. In the family under study, all affected carriers developed carotid tumours and only patient IV.1 developed jugulotympanic and vagal PGL. Multifocal tumours were observed in two members of the family under study. In patients III.2, IV.1 and V.4 and unaffected family members IV.3, IV.4, V.5 and V.6, metanephrine values were normal and 18F‐DOPA PET or thoracic/abdominal CT scans excluded the presence of PCC or other lesions not described in the manuscript.

**FIGURE 3:**
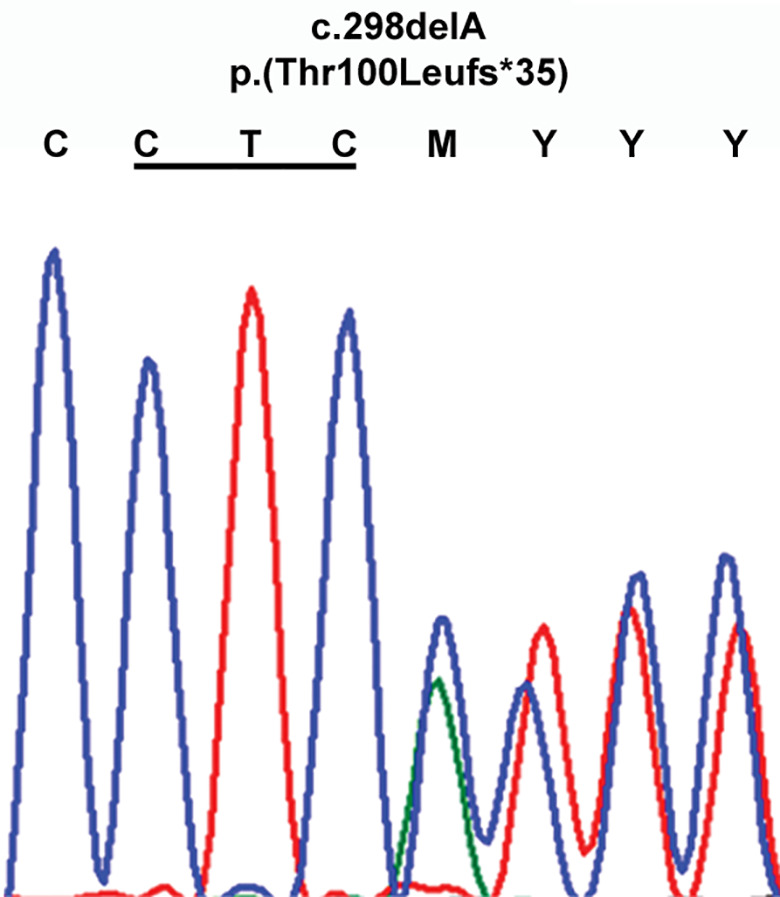
A novel heterozygous *SDHD* c.298delA frameshift variation p.(Thr100Leufs*35) identified by Sanger sequencing in the affected patient III.2. Codon p.Leu99 is underlined

Genetic screening of the grandmother III.2 for variations in the reference sequence coding regions of the *SDHD*, *SDHB* and *SDHC* genes revealed the heterozygous presence of a novel c.298delA frameshift variation (p.Thr100Leufs*35; hg19 chr 11:111,959,719) in the *SDHD* gene (figure 3). MutationTaster predicted c.298delA clinical pathogenicity as “disease causing.” The variation also affected the SDHD‐202

(c.181delA; p.Thr61Leufs*35) and SDHD‐203 (c.298delA; p.Thr100Leufs*104) isoforms.

Subsequently, all family members available to the study (n = 12) were screened for the presence of c.298delA. The transmission of tumour development followed a general paternal pattern, and offspring from female carriers of c.298delA (IV.3, IV.4, V.5, V.6) were unaffected by disease (figure 1). Although DNA from generations I and II was not available to the study, hnPGL in II.2 was documented from family history in a male who died from other causes. Assuming paternal inheritance from the generation II father to patient III.2, the 91‐year‐old living family member III.1 is therefore an asymptomatic carrier of c.298delA indicating incomplete penetrance of PGL development within the family. The absence of PGL in III.1 was confirmed by MRI (data not shown). This low penetrance had masked the genetic causes of disease in earlier generations.

## 4 DISCUSSION

### 4.1 Synopsis of key/new findings

In this study, we describe a novel *SDHD* variation (c.298delA) associated with the development of PGL with an average age of onset of 22 ± 2 years in three patients. The average age of PGL onset is 33 years with a prevalence of malignant tumour development of 4% for *SDHD* variation carriers. In 91% of hnPGL caused by *SDHD*, tumours develop primarily in the carotid and to a lesser extent in the jugular, tympanic and vagal regions. Multifocal tumours, which were present in two patients in this study, are typical for *SDHD*‐positive patients and are normally seen in 79% of *SDHD*‐positive patients.^28^

### 4.2 Transmission of SDHD variations

The c.298delA variation is not present in the Human Gene Mutation Database, dbSNP or the gnomAD database of exomes from healthy donors that should include late‐onset dominant disease alleles. Mining of *SDHD* clinically relevant variation lists from ClinVar and LOVD revealed four pathogenic variations represented in the European non‐Finnish gnomAD database (table S1).

In this study, disease was uniquely followed in a family from 2 unaffected individuals in generation I born in the 1850s through generation II born in the latter part of the 19th century and generation III (III.1 and III.2) born in the 1920s. A documented case of PGL in generation II causing the death of the patient in 1903 implies either paternal transmission with incomplete penetrance from the generation I father or atypical maternal transmission. Whereas paternal transmission can be assumed in the two sufferers in generation III, the unaffected carrier (III.1) of c.298delA has remained disease‐free for over 90 years. As maternal transmission is exceptionally rare in PGL1 and due to the evident incomplete penetrance in the case of III.1, the disease in this family seems to manifest with an uncharacteristically low penetrance.

In summary, we identify a novel c.298delA frameshift variation in *SDHD* associated with paternal transmission of hnPGL1. Genetic testing is highly recommended in hnPGL, and the unusual paternal inheritance patterns should be considered. Patients with a pathogenic SDHD variation should benefit from complete screening and lifelong follow‐up.

## CONFLICT OF INTEREST

There are no conflicts of interest.

## AUTHOR CONTRIBUTIONS

MK, TP, CS, FL, PF, KF and TL were involved in study concepts and design. MK, TP, AF, EF, FL, PF, KF and TL were involved in data acquisition and interpretation. MK, TP, KF and TL were involved in manuscript preparation and editing. All authors were involved in manuscript review.

## Supporting information

Table S1

## Data Availability

Data are available from the corresponding author on reasonable request.
