# Supplementary material for: Incomplete penetrance of a novel SDHD variation causing familial head and neck paraganglioma
Source: Clin Otolaryngol. 2021 May 5;46(5):1044–9. doi: 10.1111/coa.13782 (PMC8453574; doi:10.1111/coa.13782)
Supplement: Supplementary file 1 — Table S1 [file COA-46-1044-s001.docx]

**Supplementary table 1**

| **Coordinates**  **GRCh37(hg19)**  **Chromosome 11** | **Base change** | **Protein change** | **ClinVar ID**  **LOVD ID** | **GnomAD** |
| --- | --- | --- | --- | --- |
| 111957632 | c.1A>T | p.Met1Leu | VCV000422629 | 2/251,434 (0.000008) nFE 2/113,720  (0.00002) |
| 111957632 | c.1A>G | p.Met1Val | VCV000006911  SDHD_000021 | - |
| 111957634 | c.3G>A | p.Met1Ile | VCV000579968  SDHD_000091 | - |
| 111957634 | c.3G>C | p.Met1Ile | VCV000006906  SDHD_000015 | - |
| 111957640-1 | c.10dup | p.Leu4fs | VCV000239460 | - |
| 111957644-5 | c.13_14del | p.Trp5fs | VCV000618362 | - |
| 111957645 | c.14G>A | p.Trp5fs | VCV000006916  SDHD_000026 | - |
| 111957664 | c.33C>A | p.Cys11Ter | VCV000006915  SDHD_000027 | - |
| 111957685 | c.52+2T>G | Splice  donor | VCV000006894  SDHD_000010 | - |
| 111958580-1 | c.53dup | p.Leu19fs | VCV000279889 | - |
| 111958585 | c.57del | p.Leu20fs | VCV000006917  SDHD_000080 | - |
| 111958592 | c.64C>T | p.Arg22Ter | VCV000006903  SDHD_000012 | - |
| 111958620-1 | c.92_93TC | p.Ala33fs | VCV000006908  SDHD_000017 | - |
| 111958623 | c.95C>A | p.Ser32Ter | VCV000006901  SDHD_000008 | - |
| 111958634 | c.106C>T | p.Gln36Ter | VCV000006892  SDHD_000001 | - |
| 111958640 | c.112C>T | p.Arg38Ter | VCV000006893  SDHD_000002 | - |
| 111958652-5 | c.124_127delinsATA | p.Glu42fs | VCV000964700 | - |
| 111958657 | c.129G>A | p.Trp43Ter | VCV000006913  SDHD_000023 | - |
| 111958667 | c.139C>T | p.Gln47Ter | VCV000959874  SDHD_000169 | - |
| 111958674-5 | c.147dup | p.His50fs | VCV000231390  SDHD_000052 | - |
| 111958683 | c.155C>A | p.Ser52Ter | VCV000142068 | - |
| 111959590 | c.170-1G>T | Splice  acceptor | VCV000438434  SDHD_000031 | 1/251,124  (0.000004)  nFE 1/113,434  (0.000009) |
| 111959593 | c.173del | p.Gly58fs | VCV000239461 | - |
| 111959608-9 | c.187_188TC | p.Leu64fs | VCV000006904  SDHD_000013 | - |
| 111959624-5 | c.204_205del | p.Ser68fs | VCV000942473 | - |
| 111959662 | c.242del | p.Pro81fs | VCV000239464  SDHD_000151 | - |
| 111959663 | c.242C>T | p.Pro81Leu | VCV000006896  SDHD_000003 | 6/282,810  (0.00002)  Latino 2/35,440  (0.00006)  nFE 4/129,136  (0.00003) |
| 111959695 | c.274G>T | p.Asp92Tyr | VCV000006897  SDHD_000004 | - |
| 111959698-700 | c.278_280del | p.Tyr93del | VCV000006905  SDHD_000038 | - |
| 111959705 | c.284T>C | p.Leu95Pro | VCV000021351  SDHD_000039 | - |
| 111959716-9 | c.298_301del | p.Thr100fs | VCV000186590  SDHD_000155 | 1/251,486  (0.000004)  nFE 1/113,762  (0.000009) |
| 111959725 | c.304C>A | p.His102Asn | VCV000185719 | - |
| 111959726 | c.305A>T | p.His102Leu | VCV000006898  SDHD_000005 | - |
| 111959735 | c.314G>A | p.Trp105Ter | VCV000428939 | - |
| 111959736 | c.314+1G>T | Splice  donor | VCV000480808  SDHD_000087 | - |
| 111959736 | c.314+1G>A | Splice  donor | VCV000438436  SDHD_000060 | - |
| 111965529 | c.315G>A | p.Trp105Ter | VCV000412497 | - |
| 111965539 | c.325C>T | p.Gln109Ter | VCV000412498  SDHD_000046 | - |
| 111965549-50 | c.336dup | p.Asp113Ter | VCV000547770 | - |
| 111965548-51 | c.337_340del | p.Asp113fs | VCV000006912  SDHD_000022 | - |
| 111965551-2 | c.337_338insT | p.Asp113fs | VCV000006899  SDHD_000006 | - |
| 111965555 | c.341A>G | p.Tyr114Cys | VCV000006900  SDHD_000007 | - |
| 111965556 | c.342T>A | p.Tyr114Ter | VCV000438437  SDHD_000083 | - |
| 111965575 | c.361C>T | p.Gln121Ter | VCV000239470  SDHD_000047 | - |
| 111965593 | c.381del | p.Leu128fs | VCV000438439  SDHD_000029 | - |
| 111965596-7 | c.383_386dup | p.Leu129fs | VCV000951786 | - |
| 111965606 | c.394del | p.Ser132fs | VCV000412505 | - |
| 111965630 | c.416T>C | p.Leu139Pro | VCV000006907  SDHD_000016 | - |
| 111965655 | c.443del | p.Gly148fs | VCV000006902  SDHD_000049 | - |
| 111965677 | c.463del | p.Met155fs | VCV000006910  SDHD_000020 | - |

**Supplementary table 1:** SDHD, duplications, small deletions, missense, nonsense, splice donor and splice acceptor variations listed in ClinVar and the Leiden Open Variation Database (LOVD) to cause pheochromocytoma and paraganglioma 1. Global allele frequencies, from gnomAD v2.1.1 are listed. Non-Finnish European (nFE).
